# BreCML: identifying breast cancer cell state in scRNA-seq via machine learning

**DOI:** 10.3389/fmed.2024.1482726

**Published:** 2024-11-06

**Authors:** Shanbao Ke, Yuxuan Huang, Dong Wang, Qiang Jiang, Zhangyang Luo, Baiyu Li, Danfang Yan, Jianwei Zhou

**Affiliations:** ^1^Department of Oncology, Henan Provincial People’s Hospital, Zhengzhou University People’s Hospital, Zhengzhou, China; ^2^Department of Neuroscience in the Behavioral Sciences, Duke University and Duke Kunshan University, Suzhou, China; ^3^Pudong Institute for Health Development, Shanghai, China; ^4^Department of Radiation Oncology, The First Affiliated Hospital, College of Medicine, Zhejiang University, Hangzhou, China

**Keywords:** breast cancer, machine learning, scRNA-seq, cell subpopulations, feature selection

## Abstract

Breast cancer is a prevalent malignancy and one of the leading causes of cancer-related mortality among women worldwide. This disease typically manifests through the abnormal proliferation and dissemination of malignant cells within breast tissue. Current diagnostic and therapeutic strategies face significant challenges in accurately identifying and localizing specific subtypes of breast cancer. In this study, we developed a novel machine learning-based predictor, BreCML, designed to accurately classify subpopulations of breast cancer cells and their associated marker genes. BreCML exhibits outstanding predictive performance, achieving an accuracy of 98.92% on the training dataset. Utilizing the XGBoost algorithm, BreCML demonstrates superior accuracy (98.67%), precision (99.15%), recall (99.49%), and F1-score (99.79%) on the test dataset. Through the application of machine learning and feature selection techniques, BreCML successfully identified new key genes. This predictor not only serves as a powerful tool for assessing breast cancer cellular status but also offers a rapid and efficient means to uncover potential biomarkers, providing critical insights for precision medicine and therapeutic strategies.

## Introduction

Breast cancer is considered the most common malignant tumor worldwide and is one of the leading causes of cancer-related deaths among women globally (1, 2). The incidence of breast cancer is influenced by multiple factors, including age, genetic background, and reproductive history and so on. Long-term exposure to ovarian steroids is widely recognized as a risk factor for breast cancer in women, and studies have shown a significant correlation between the total number of menstrual cycles and the risk of breast cancer (3–5). Breast cancer is commonly described as an “immunologically cold” tumor (6), characterized by a low mutation count, limited immune cell infiltration, and immunosuppressive features in the tumor microenvironment (7).

A detailed exploration of the cellular subtypes of breast cancer is crucial for developing more precise clinical treatment protocols and for advancing pathophysiological research. The genetic heterogeneity of breast cancer has been confirmed at a single-cell resolution, a process dependent on high-density genome coverage (8). With the ongoing advancement of single-cell sequencing technology, we are now able to explore the cellular heterogeneity of this cancer at an even higher resolution.

Through single-cell transcriptomic analysis, Chung et al. (9) explored the heterogeneity of tumor cells and their neighboring immune and stromal cells, revealing significant heterogeneity both within the tumor and among immune cells. Jang et al. (10) utilized single-cell RNA sequencing (scRNA-seq) technology to analyze the transcriptional and mutational features of breast cancer and immune cells. They identified high PD-L1 expression and significant microsatellite instability in radioresistant cells, along with complex interactions at immune checkpoints. These findings provide potential biomarkers and therapeutic strategies for immunotherapy and radiation therapy tailored to different subtypes of breast cancer. Liu et al. (11) combined scRNA-seq with spatial transcriptomics to analyze the cell populations and their spatial distribution in breast cancer. They identified subpopulations of malignant cells, revealing their locations and the relationships with patient survival and therapeutic responses, which provided new insights into the heterogeneity of breast cancer and potential personalized treatment strategies. Ding et al. (12) discussed the application of scRNA-seq in breast cancer research. Through technological advancements, scRNA-seq has revealed cellular heterogeneity in the tumor microenvironment and identified disease-related rare cell types. This technique has demonstrated its potential in classifying breast cancer subtypes, recognizing immune cell subgroups, and identifying therapeutic targets, thereby facilitating the development of personalized treatment strategies.

**Figure 1:**
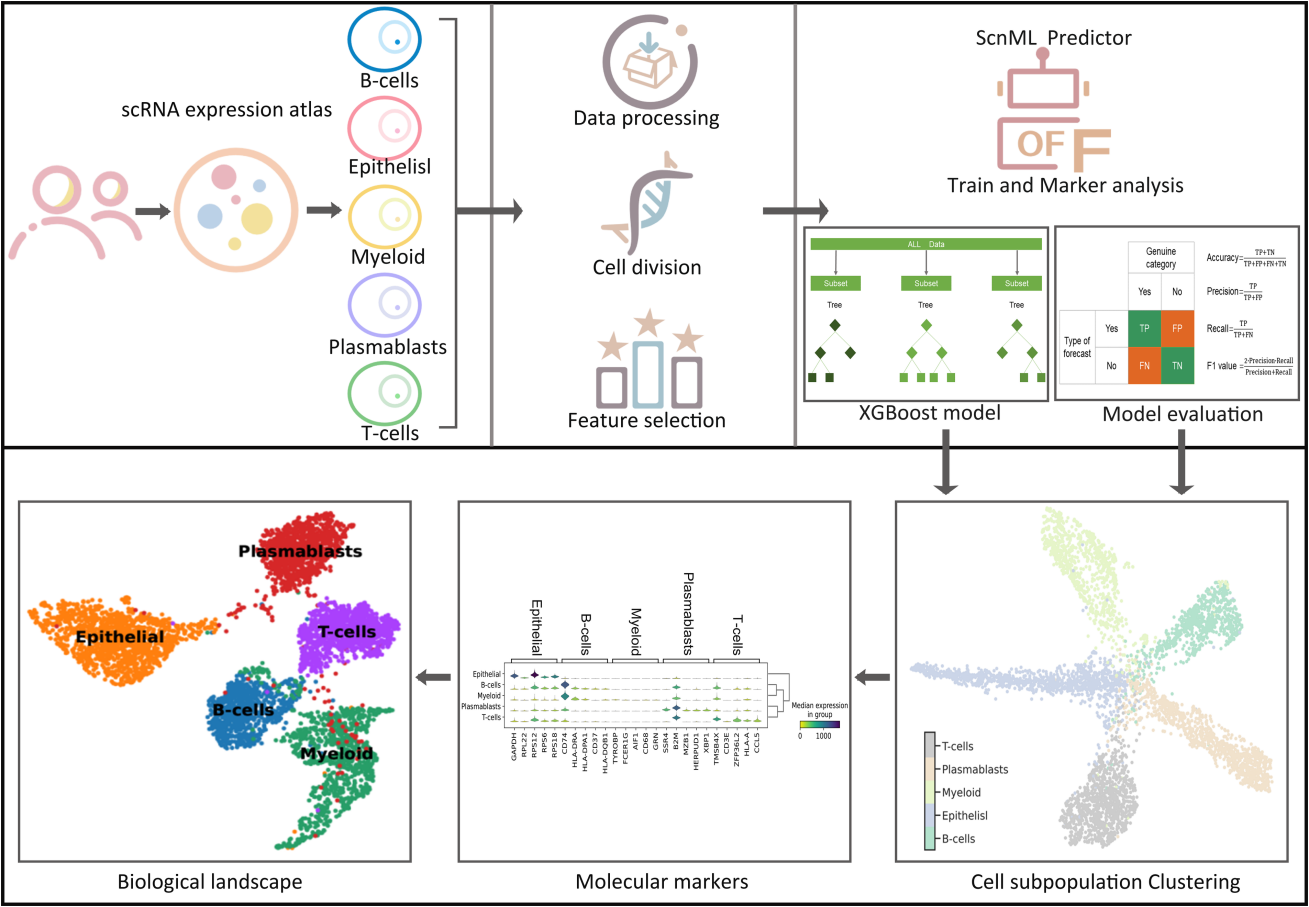
The workflow of constructing BreCML.

Although existing research technologies in the field of breast cancer are relatively advanced, manual methods remain time-consuming and labor-intensive when it comes to mining marker genes and identifying cell subgroups. Consequently, there is an urgent need for the development of computational methods to assist researchers in efficiently identifying breast cancer cell subgroups and thoroughly exploring their potential marker genes. To address these challenges, we introduced a computational framework named BreCML (Figure 1). This framework is designed to identify biomarkers within breast cancer cell subpopulations and infer their cellular developmental stages, thereby enhancing the accuracy and depth of research in this area. To achieve optimal predictive modeling results, we employed a combined feature selection and incremental feature selection (IFS) strategy. This strategy incorporates the use of four fundamental classification methods: K-nearest neighbors (KNN), extreme gradient boosting (XGBoost), support vector machine (SVM), and random forest classification (RFC).

## Results

### Identify important genes by BreCML

**Figure 2:**
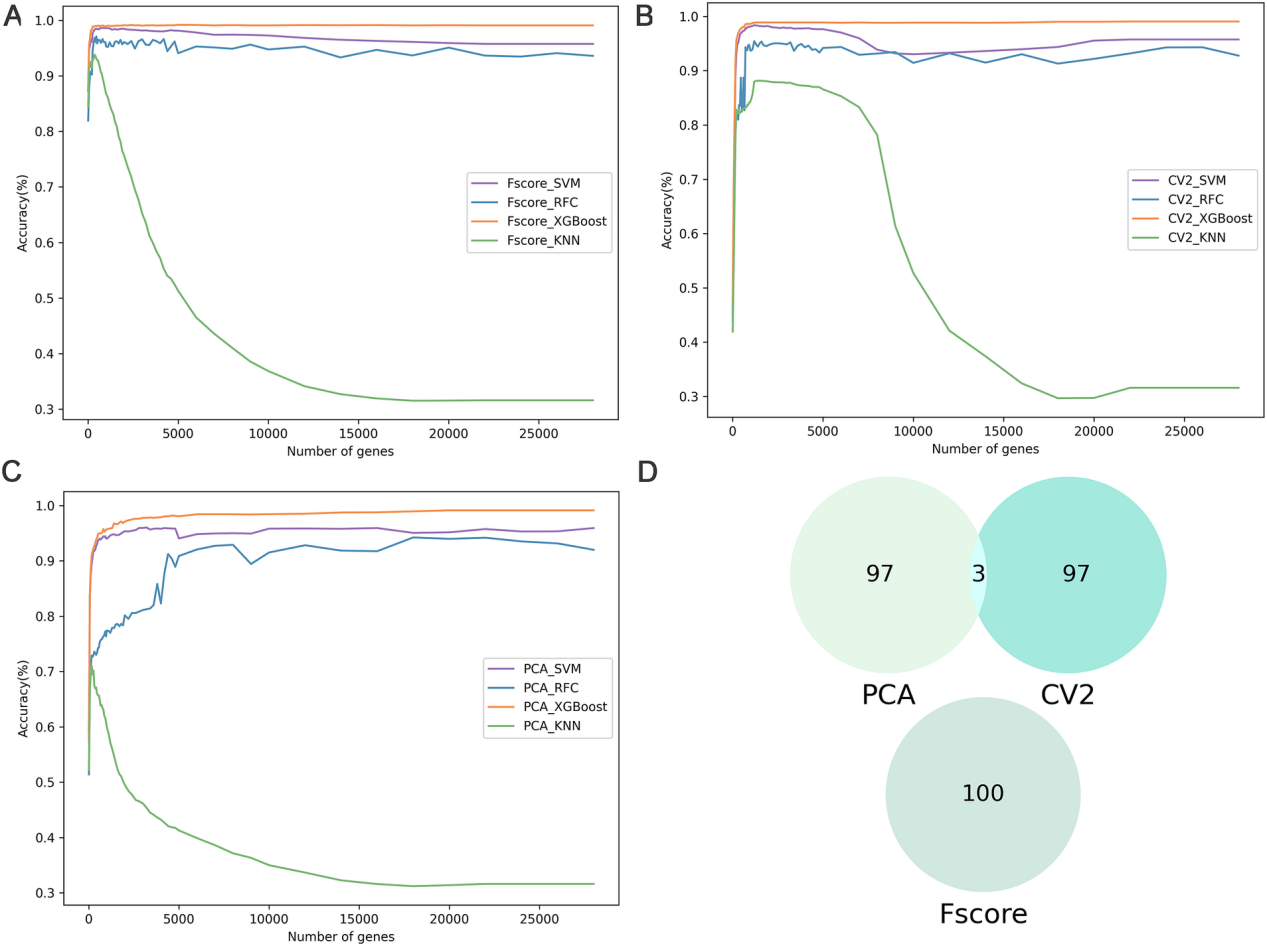
The results of feature selection. **(A–C)** The IFS curves show the performance of three feature selections (*F*-score, CV^2^, and PCA) and the four classifiers in different gene subsets. **(D)** Comparative Venn diagram of the top 100 genes in *F*-score, CV^2^, and PCA.

To identify key genes associated with subpopulations of breast cancer cells, we employed three feature selection methods *F*-score, coefficient of variation squared (CV^2^), and principal component analysis (PCA) to evaluate the significance of 29,733 genes and rank them according to their contribution (Figures 2A–C). Genes with importance scores less than or equal to zero were excluded from further analysis. The CV^2^, PCA, and *F*-score extracted 22,000 important genes. Subsequently, machine learning models combined with incremental feature selection (IFS) were utilized to identify the optimal subset of genes. Using five-fold cross-validation, the machine learning models (SVM, RFC, XGBoost, and KNN) were trained with single-cell gene expression matrices as input features.

The analysis of the training dataset showed that the combination of *F*-score and XGBoost model (BreCML) using the top 360 genes achieved the best predictive performance, successfully classifying breast cancer cell subpopulations with 98.92% accuracy (Supplementary Table S3). Notably, significant predictive performance was also achieved when the four machine learning models were combined with PCA. However, BreCML uses only 360 feature genes, while XGBoost combined with PCA uses 20,000 feature genes, meaning that the complexity of the model was greatly reduced. Therefore, BreCML was selected as the classifier by us. To prevent the feature selection methods from exhibiting similar scoring preferences, we compared the top 100 genes ranked by each feature selection method. As demonstrated in Figure 2D, the top 100 genes selected by PCA, CV^2^, and *F*-score exhibit minimal overlap, thereby validating the distinct effectiveness of each feature selection method. These findings underscore the utility of combining diverse feature selection techniques to enhance the robustness and accuracy of predictive models in breast cancer research.

### BreCML performance on test dataset

**Figure 3:**
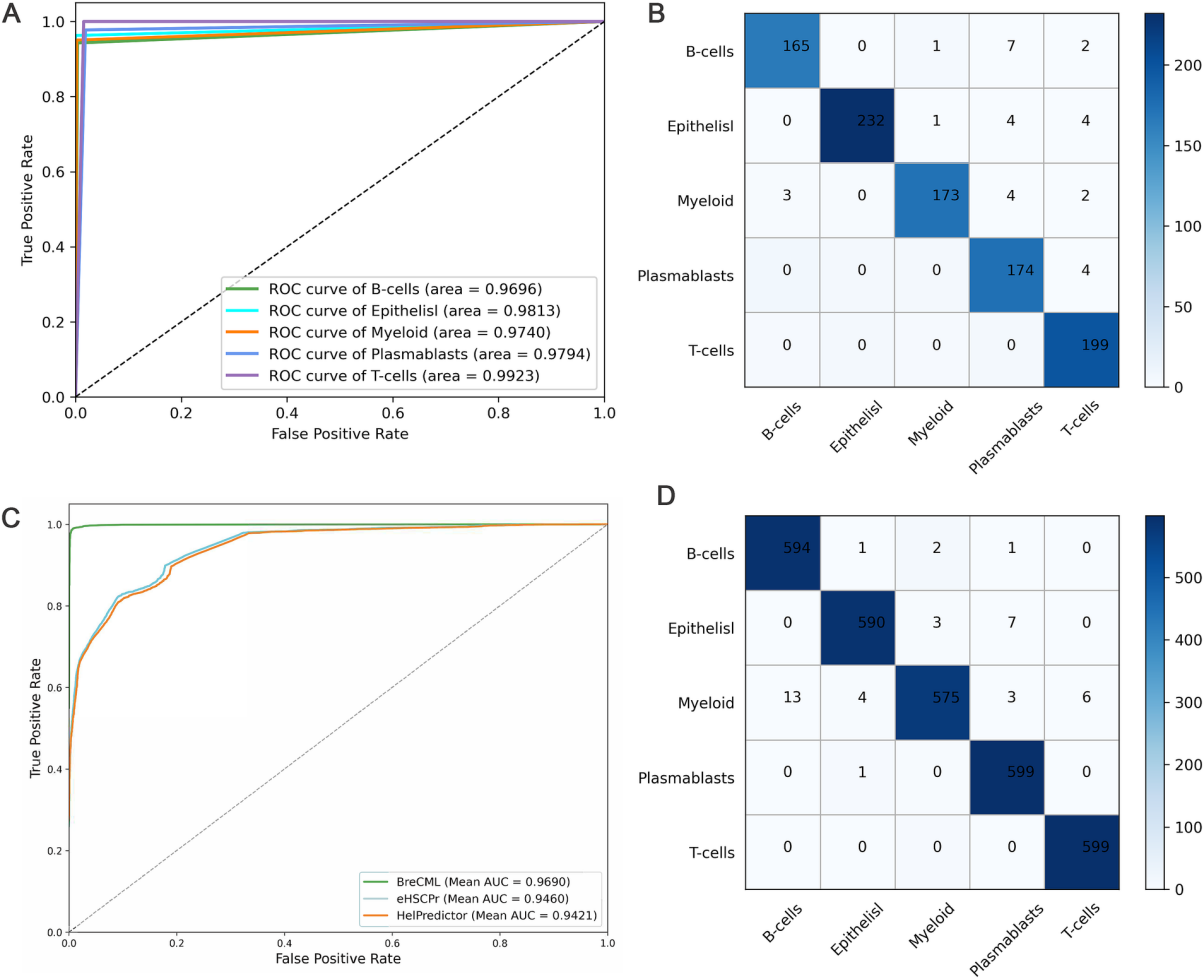
Predictive performance of BreCML. **(A)** ROC curves for BreCML on test dataset. **(B)** The confusion matrix shows the accuracy of BreCML using 360 genes from BreCML algorithm on test dataset. **(C)** ROC curves and AUC show the performance of the BreCML with other state-of-the-art methods on an independent dataset. **(D)** Based on the BreCML optimal gene set, the confusion matrix of BreCML on the independent dataset.

BreCML demonstrated exceptional performance on the test dataset, achieving outstanding results across several key metrics: accuracy of 98.67%, precision of 99.15%, recall of 99.49%, and F1-score of 99.79% (Table 1). To further evaluate the model’s effectiveness, we assessed its predictive capabilities using Receiver Operating Characteristic (ROC) curves and confusion matrices. The ROC analysis revealed an impressive area under the curve (AUC) of 0.97 for the BreCML model, as shown in Figure 3A. Additionally, the confusion matrix provided a detailed breakdown of the model’s performance across different breast cancer subgroups, highlighting a notably low misclassification rate (Figure 3B). This strong performance underscores the robustness and reliability of the BreCML model in clinical diagnostics.

**Table 1:** Performance comparison of different algorithms and feature selection strategies (test dataset).

Method	Feature selection	No. of feature	Accuracy	Precision	Recall	F1-measure
KNN	*F*-score	360	94.78	94.98	95.54	95.79
RFC	*F*-score	360	96.72	97.89	97.54	96.97
SVM	*F*-score	860	99.08	99.78	99.68	99.82
XGBoost	*F*-score	360	98.67	99.15	99.49	99.79
KNN	CV^2^	1,500	88.92	89.22	89.74	89.18
RFC	CV^2^	1,200	95.18	95.73	95.79	96.39
SVM	CV^2^	1,200	98.15	98.76	99.27	98.94
XGBoost	CV^2^	22,000	98.97	99.12	99.67	99.81
KNN	PCA	160	70.67	71.49	70.91	71.59
RFC	PCA	18,000	93.44	94.78	94.16	93.87
SVM	PCA	3,200	95.59	96.48	96.29	95.91
XGBoost	PCA	20,000	99.18	99.94	99.87	99.64

### Predictive performance of BreCML on an independent test set

To evaluate the robustness of the proposed BreCML for breast cancer cell subpopulation prediction, we assessed the performance of BreCML in an independent dataset and compared it with two state-of-the-art methods: eHSCPr, and HelPredictor. To ensure a fair comparison, these models were executed and evaluated using the same independent test set containing 360 genes. As shown in Table 2, BreCML achieved the best performance among all of the tested methods, with an accuracy of 94.78%, precision of 94.98%, recall of 95.54%, and F1-measure of 95.79%. Specifically, compared to other existing methods, the accuracy of our method is higher by 4.66 to 6.53%. The AUC of the three methods is shown in Figure 3C, and the AUC of BreCML is 0.97, which is outperformed by the other prediction models. Furthermore, the confusion matrix further validated the predictive performance of the model for each cell subpopulation, and the low misclassification rate demonstrated the power of the BreCML model (Figure 3D). Therefore, we conclude that our method is more effective than eHSCPr, and HelPredictor in predicting breast cancer cell subpopulations.

**Table 2:** Performance comparison between BreCML and the other algorithms (independent dataset).

Method	Accuracy	Precision	Recall	F1-measure
BreCML	94.78	94.98	95.54	95.79
eHSCPr	88.25	87.29	87.16	85.71
HelPredictor	90.12	88.02	88.32	96.33

### Expression analysis of the BreCML gene set

**Figure 4:**
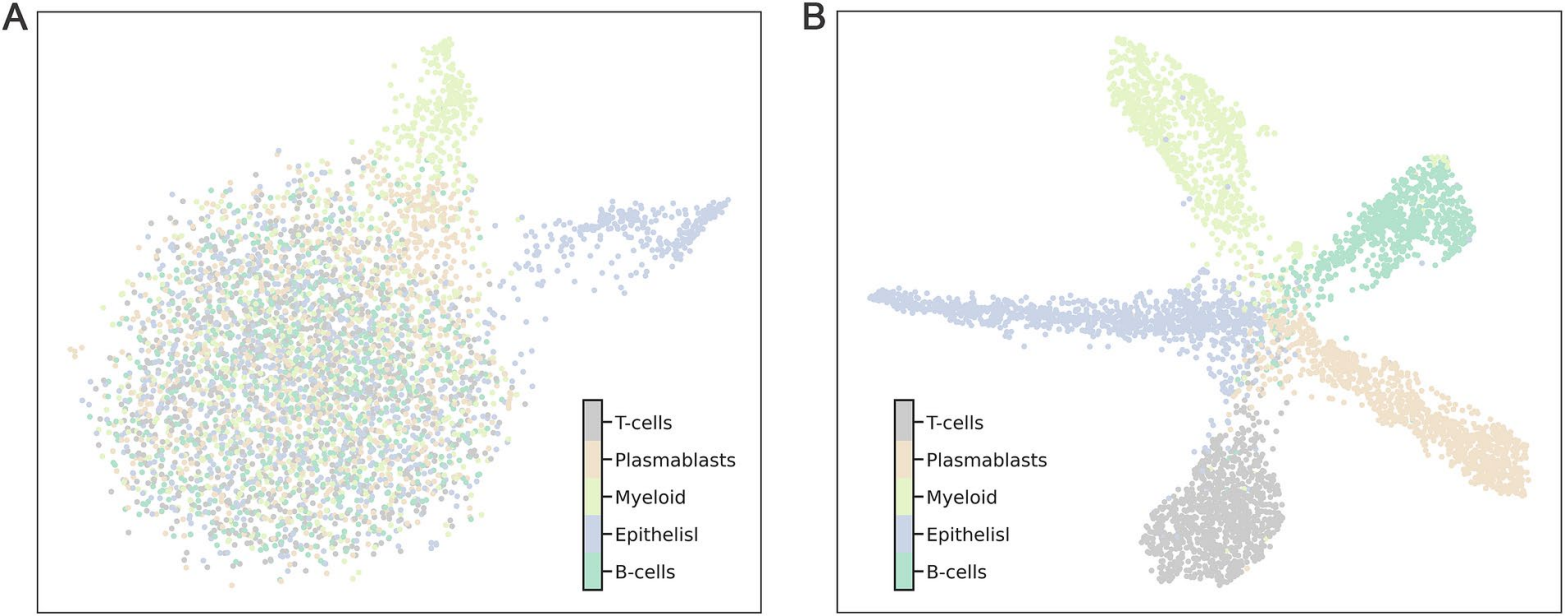
The clustering effect on 4,874 cells was evaluated using 110 marker genes and all genes (**A** represents all genes, **B** represents the 110 marker genes). Each point represents a sample in the dataset, and different categories of samples are given different colors.

Further analysis was performed using Uniform Manifold Approximation and Projection (UMAP) on 4,874 single cells to evaluate the comparative performance of the 360 selected genes against the entire gene set. The results demonstrated that the 360 selected genes significantly outperformed the full gene set in terms of clustering efficiency and specificity. When clustering was conducted using all genes, samples from different categories were almost entirely intermingled, resulting in poor classification outcomes (Figure 4A). In contrast, the application of the top 360 genes produced a clear and distinct distribution of cell subpopulations (Figure 4B). This enhanced clustering not only improved the visual differentiation of categories but also underscored the effectiveness of selecting key marker genes for precise subpopulation identification.

**Figure 5:**
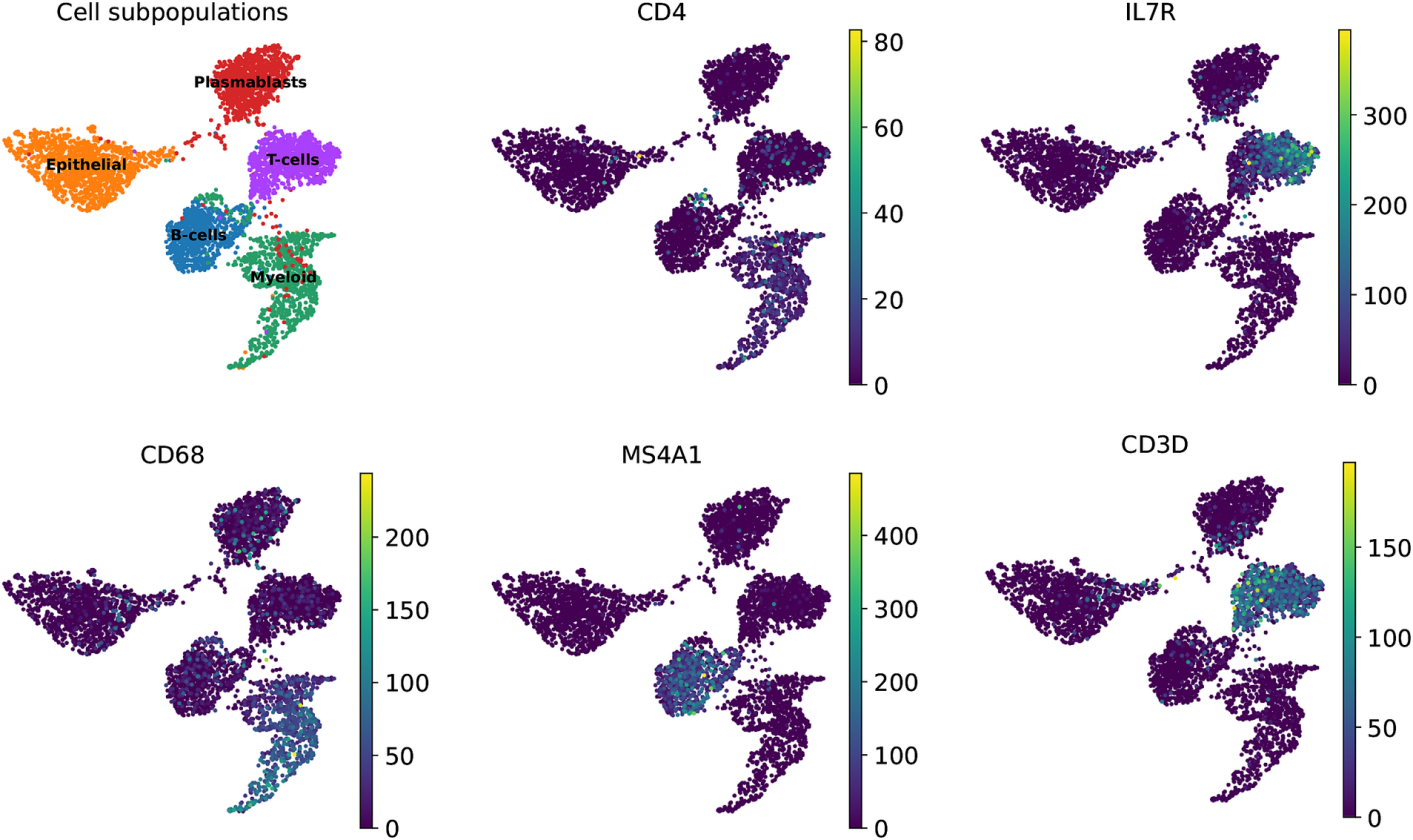
UMAP shows marker genes for human breast cancer cell fate determination.

Additionally, we explored the representation of the 360 marker genes across the biological landscape, identifying several key genes that serve as robust markers for specific cell types within the immune system. Notably, genes such as *CD4*, *IL7R*, and *CD3D* were found to be highly expressed in T-cells, underscoring their importance in cellular immunity functions. Similarly, *CD68* was predominantly expressed in myeloid cells, while *MS4A1* was identified as a characteristic gene of a B-cell subpopulation (Figure 5). These genes have undergone rigorous validation, and their expression patterns have been corroborated by extensive literature, highlighting their biological relevance and utility in cellular characterization.

**Figure 6:**
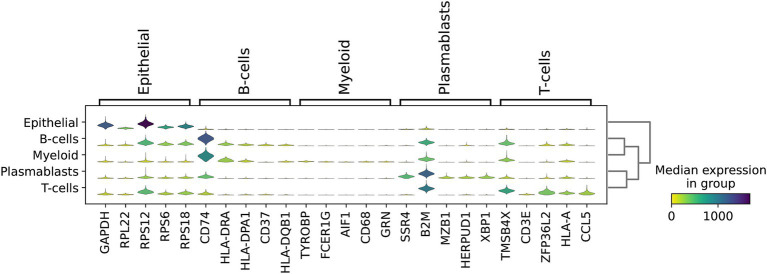
High expression marker genes screened by Scanpy.

Using multiple genes to characterize cellular subpopulations significantly enhances accuracy. For instance, *GAPDH*, *RPL22*, *RPS12*, *RPS6*, and *RPS18* were crucial in identifying epithelial subpopulations. Similarly, *CD74*, *HLA-DRA*, and *HLA-DPA1* exhibited high expression levels in B-cells, while *SSR4*, *B2M*, *MZB1*, *HERPUD1*, and *XBP1* were prominently expressed in plasmablasts. Additionally, *TMSB4X*, *ZFP36L2*, *HLA-A*, and *CCL5* were highly expressed in T-cells (Figure 6). This multi-gene approach not only improves the precision of cell type identification but also provides a more comprehensive understanding of the molecular signatures associated with different cellular subpopulations.

## Conclusion

Breast cancer is one of the most prevalent malignant tumors in women, and single-cell RNA sequencing technology plays a crucial role in uncovering its tumor heterogeneity and developmental mechanisms. In this study, we utilized single-cell sequencing technology to conduct an in-depth analysis of various cell subpopulations and their molecular characteristics within breast cancer tissues. We designed and developed a machine learning-based prediction model, BreCML, which demonstrated exceptional performance in predicting breast cancer cell subpopulations. This was evidenced by the results from an independent dataset, where BreCML achieved an accuracy of 98.92% and an ROC of 0.97. BreCML addresses the computational inefficiency and overfitting issues typically associated with the high-dimensional feature space, thereby significantly enhancing the prediction accuracy and robustness of the model. Moreover, by analyzing the BreCML model, we identified a set of key genes that can serve as biomarkers for breast cancer cell subpopulations. These markers hold promise for providing new breakthroughs in early diagnosis and personalized treatment.

However, this study is certainly not without its limitations. A major limitation is the small sample size, and collaborative efforts in data collection may help to improve the model. Despite this potential limitation of the current study, our work provides a resource for studying biomarkers of breast cancer cell subpopulations at single-cell resolution. This not only enhances our understanding of the molecular mechanisms underlying breast cancer but also provides a vital molecular tool for assessing the complexity of breast cancer cell subpopulations, with profound implications for future clinical research.

## Methods and materials

### Dataset construction and preprocessing

Single-cell transcriptome data for human breast cancer were obtained from the National Center for Biotechnology Information (GSE176078) and include 4,874 cells (13). GSE176078 is one of the most comprehensive scRNA-seq dataset specifically focused on breast cancer, including a wide variety of breast cancer subpopulations. This dataset offers detailed transcriptional profiles of thousands of individual cells from multiple patients, making it highly suitable for studying intratumoral heterogeneity, identifying distinct cell subpopulations, and exploring cell-specific biomarkers. The BCL files were demultiplexed and aligned to the GRCh38 reference genome using Cell Ranger Single Cell software v2.0 (10× Genomics). Cell filtering was performed with the EmptyDrops method from the DropletUtils package v1.2.2, applying additional criteria: cells with more than 200 genes and 250 unique molecular identifiers, and a mitochondrial gene percentage below 20%. The dataset included five different cell subpopulations: B-cells (773), Cancer Epithelisl (1,184), Myeloid (897), Plasmablasts (1,020), and T-cells (1,000). The dataset was split into a training dataset and a test dataset at a 8:2 ratio. More dataset details are provided in the Supplementary Table S1. The Python packages Numpy (version 1.21.6), Pandas (version 1.3.5) and Scanpy (version 1.9.1) were used to read and process the data.

To further validate the robustness of BreCML, we collected single-cell transcriptome data of breast cancer from Wu et al. (14) in the NCBI database (GSE158677). The dataset also included five different cell subpopulations: B cells (598), Cancer epithelial cells (600), myeloid cells (601), Plasmablasts cells (600), and T cells (599). This dataset was used as an independent test set to evaluate the BreCML performance (Supplementary Table S2).

### Biological analysis and visualization

In this study, we performed an extensive analysis to evaluate the predictive capability of 360 marker genes in identifying cell subpopulations. For the clustering analysis in Figure 4, UMAP visualization was executed using the python package umap-learn (version 0.3.9), with all settings maintained at default values. For the clustering analysis in Figure 5, we used the Preprocessing and clustering module in Scanpy (version 1.9.1), which facilitated to identify specific subpopulations of cells associated with these marker genes; default parameters were used throughout. Pearson correlation analysis was conducted on five distinct human breast cancer cell populations, based on the expression profiles of the 360 marker genes, using Pandas (version 1.4.4).

### Principal component analysis

Feature-scML is a scalable and friendly toolkit that allows users to comprehensively score and rank each feature in scRNA-seq data. The PCA module of Feature-scML was used to assess the feature importance of each gene. The source code is available at https://github.com/liameihao/Feature-scML.

### *F*-score algorithm

The *F*-score can be used to measure the degree of differentiation of features in different categories and has been shown to be a simple and effective method for feature selection. This method significantly improves the interpretability and classification performance of the model while reducing the bias (15). The *F*-score of the *i*th feature is defined as (16, 17):


(1) $$Fi=\frac{{\left({\bar{X_{i}}}^{\left(+\right)}-\bar{X_{i}}\right)}^{2}+{\left({\bar{X_{i}}}^{\left(-\right)}-\bar{X_{i}}\right)}^{2}}{\frac{1}{n_{+}-1}\sum_{k=1}^{n+}{\left({\bar{X}}_{k,i}^{\left(+\right)}-{\bar{X_{i}}}^{\left(+\right)}\right)}^{2}+\frac{1}{n-1}\sum_{k=1}^{n-}{\left(X_{k,i}^{\left(-1\right)}-{\bar{X_{i}}}^{\left(-\right)}\right)}^{2}}$$

where $\bar{X_{i}}$ represents the average of the ith feature of the whole ${\bar{X_{i}}}^{\left(+\right)}$ is the number of positive samples, ${\bar{X_{i}}}^{\left(-\right)}$ is the number of negative samples. ${\bar{X}}_{k,i}^{\left(+\right)}$ , ${\bar{X}}_{i,i}^{\left(-\right)}$ are the ith feature of the *k*th positive and negative instances, respectively. The larger the *F*-score value, the stronger the distinguishing degree of the feature among different categories.

### Squared coefficient of variation

The squared coefficient of variation (CV^2^) is a quantitative statistical method for quantifying technical variation at the gene level and assessing variability in cell biology, and is widely used in the field of single-cell experiments (18). The CV^2^ method operates by calculating the squares of the coefficients of variation and curve-fitting the observations using the generalized linear model (GLM) in the R package statmod,


(2) $${CV}^{2}=\frac{a1}{\mu }+\alpha 0$$

### Extreme gradient boosting

Extreme gradient boosting (XGBoost) is recognized as an exceedingly complex and efficient machine learning algorithm, widely acknowledged for its outstanding performance in predictive modeling competitions (19). XGBoost attracts significant attention primarily due to its efficiency and effectiveness demonstrated in various competitive settings. The algorithm operates by sequentially constructing a series of decision trees, each designed to correct the errors of its predecessor. This approach allows the model to capture complex patterns in the data, thereby enhancing predictive accuracy. One major advantage of XGBoost is its ability to quickly and accurately process large datasets, making it an ideal tool for our research. Furthermore, compared to other models such as KNN and SVM, XGBoost also offers a direct method for assessing the importance of each input variable.

### Model construction of BreCML

During the exploratory data analysis, it was crucial to identify key relationships and assign appropriate weights to features to filter out less relevant or weaker information. We employed three feature selection techniques PCA, CV^2^, and *F*-score—to evaluate and rank the importance of genes in descending order. Genes with weights equal to or below zero were excluded from further analysis. The sorted gene expression profiles of breast cancer cell subpopulations served as input features for training machine learning models. Utilizing the incremental feature selection (IFS) strategy, we formed 12 combinations by integrating the three feature selection methods with four machine learning models: KNN, XGBoost, SVM, and RFC. Grid search was used to determine the optimal parameters for each combination. The optimal gene set for each combination was identified when the accuracy no longer showed improvement with the addition of more genes. Ultimately, the combination of *F*-score and XGBoost proved to be the most effective and was employed to develop the BreCML model.

### Model evaluation

The four classic metrics were used to quantify the performance of the model predictions, namely, the accuracy (Acc), recall (Re), precision (Pre), and F1 measure (F1), defined as (20–28):


(3) $$\mathrm{Accuracy}=\frac{TP+TN}{TP+TN+FP+FN}$$

(4) $$\mathrm{Recall}=\frac{TP}{TP+FN}$$

(5) $$\mathrm{Precision}=\frac{TP}{TP+FP}$$

(6) $$F1\;\mathrm{measure}=\frac{2*\left(\mathrm{precision}*\mathrm{recall}\right)}{\mathrm{precision}+\mathrm{recall}}$$

where TP, TN, FP, and FN represent the numbers of true positives, true negatives, false positives and false negatives, respectively. In addition, the ROC curve was used to evaluate the performance of the BreCML (29–32).

## Data Availability

Single-cell transcriptome data for human breast cancer were obtained from the National Center for Biotechnology Information (GSE176078 and GSE158677). The processed data from this study are available at https://www.jianguoyun.com/p/DV0r_W0QsKH7DBictdkFIAA.
