# Supplementary material for: BreCML: identifying breast cancer cell state in scRNA-seq via machine learning
Source: Front Med (Lausanne). 2024 Nov 6;11:1482726. doi: 10.3389/fmed.2024.1482726 (PMC11579858; doi:10.3389/fmed.2024.1482726)
Supplement: Supplementary file 1 [file Table_1.DOCX]

**BreCML: identifying breast cancer cell state in scRNA-seq via machine learning**

[Shanbao Ke](https://pubmed.ncbi.nlm.nih.gov/?term=Ke+S&cauthor_id=29745068)^1,†^, Yuxuan Huang^3,†^, Dong Wang^4,†^, Qiang Jiang^1^, Luo Zhanyang^4^, Baiyu Li^1^, Danfang Yan^2^, Jianwei Zhou^1,*^

1 Department of Oncology, Henan Provincial People's Hospital, Zhengzhou University People's Hospital, Zhengzhou, 450003, China.

2 Department of Radiation Oncology, The First Affiliated Hospital, College of Medicine, Zhejiang University, Hangzhou, 310003, China.

3 Department of Neuroscience in the Behavioral Sciences, Duke University and Duke Kunshan University, Suzhou, China

4 Pudong Institute for Health Development, Shanghai, 200137, China.

† These authors contributed equally to this work.

* Corresponding authors: Jianwei Zhou, Department of Oncology, Henan Provincial People's Hospital; Zhengzhou University People's Hospital, Zhengzhou, China. Email: 18037790277@163.com.

**Supplementary Table 1** The composition of data on human breast cancer cell subpopulations (GSE176078).

| Cell type | Number of cells(train) | Number of cells(test) | Total number |
| --- | --- | --- | --- |
| B-cells | 598 | 175 | 773 |
| VCancer Epithelisl | 943 | 241 | 1184 |
| VMyeloid | 715 | 182 | 897 |
| Plasmablasts | 842 | 178 | 1020 |
| T-cells | 801 | 199 | 1000 |

**Supplementary Table 2** The composition of data on human breast cancer cell subpopulations (GSE158677, Independent dataset).

| Cell type | Total number |
| --- | --- |
| B-cells | 598 |
| VCancer Epithelisl | 600 |
| VMyeloid | 601 |
| Plasmablasts | 600 |
| T-cells | 599 |

**Supplementary Table 3** Performance comparison of different algorithms and feature selection strategies (Train dataset, five-fold cross-validation).

| Method | Feature selection | No.of feature | Train (mean ± standard error) |
| --- | --- | --- | --- |
| KNN | F-score | 360 | 93.52 ± 0.013 |
| RFC | F-score | 360 | 95.98 ± 0.025 |
| SVM | F-score | 860 | 97.02 ± 0.019 |
| XGBoost | F-score | 360 | 98.88 ± 0.037 |
| KNN | CV2 | 1500 | 88.56 ± 0.022 |
| RFC | CV2 | 1200 | 94.95 ± 0.038 |
| SVM | CV2 | 1200 | 98.01 ± 0.033 |
| XGBoost | CV2 | 22000 | 98.85 ± 0.025 |
| KNN | PCA | 160 | 73.87 ± 0.026 |
| RFC | PCA | 18000 | 94.39 ± 0.048 |
| SVM | PCA | 3200 | 96.88 ± 0.031 |
| XGBoost | PCA | 20000 | 98.39 ± 0.042 |
